# Association of Upon-Diagnosis Blood Eosinophilic Count with Frequency and Severity of Annual Exacerbation in Chronic Obstructive Pulmonary Disease: A Prospective Longitudinal Analysis

**DOI:** 10.1155/2023/8678702

**Published:** 2023-04-26

**Authors:** Arda Kiani, Fatemehsadat Rahimi, Siamak Afaghi, Maryam Paat, Mohammad Varharam, Mehdi Kazempour Dizaji, Maryam Dastoorpoor, Atefeh Abedini

**Affiliations:** ^1^Chronic Respiratory Disease Research Center, National Research Institute of Tuberculosis and Lung Diseases, Masih Daneshvari Hospital, Shahid Beheshti University of Medical Sciences, Tehran, Iran; ^2^Prevention of Metabolic Disorders Research Center, Research Institute for Endocrine Sciences, Shahid Beheshti University of Medical Sciences, Tehran, Iran; ^3^Department of Pulmonary Medicine, Clinical Research and Development Center, Masih Daneshvari Hospital, Shahid Beheshti University of Medical Sciences, Tehran, Iran; ^4^Mycobacteriology Research Center, National Research Institute of Tuberculosis and Lung Disease, Shahid Beheshti University of Medical Sciences, Tehran, Iran; ^5^Biostatistics Department, Mycobacteriology Research Centre, National Research Institute of Tuberculosis and and Lung Diseases, Shahid Beheshti University of Medical Sciences, Tehran, Iran; ^6^Department of Biostatistics and Epidemiology, Air Pollution and Respiratory Diseases Research Center, Ahvaz Jundishapur University of Medical Science, Ahvaz, Iran

## Abstract

**Introduction:**

There is a controversy regarding the relationship between blood eosinophil count and COPD exacerbation. We aimed to determine whether peripheral eosinophils upon COPD diagnosis could affect the frequency and severity of annual acute exacerbation of COPD (AECOPD).

**Methods:**

This prospective study was conducted on 973 newly diagnosed COPD patients who were under 1-year follow-up in a pulmonology center in Iran. The Cox proportional model, polynomial regression, and receiver operator characteristic curves were conducted to evaluate the impact of the eosinophil levels on AECOPD. A linear regression model was conducted to evaluate the continuous association of eosinophilic count with AECOPDs.

**Results:**

Patients with eosinophil >200 cells/microliter were higher pack-year smokers with more pulmonary hypertension prevalence compared to COPD patients with <200 cells/microliter. There was a positive correlation between the eosinophilic count and the frequency of AECOPDs. Eosinophil >900 cells/microliter and eosinophil >600 cells/microliter had a sensitivity of 71.1% and 64.3%, respectively, in predicting the occurrence of more than one AECOPD. Eosinophilic count cutoff of 800 cells/microliter had the highest Youden index with sensitivity and specificity of 80.2% and 76.6%, respectively, for incident AECOPD in newly diagnosed patients. Using a linear model, increasing 180 cells/microliter in serum eosinophils was associated with further exacerbation. Evaluating gender, BMI, smoking pack-year, FEV1/FVC, CAT score, GOLD score, pulmonary hypertension, annual influenza, pneumococcal vaccinations, leukocytosis, and blood eosinophils, only blood eosinophils (hazard ratio (HR) = 1.44; 95% confidence interval = 1.33–2.15; *p* value = 0.03) and GOLD score (HR = 1.19; 95% CI = 1.30–1.52; *p* value = 0.03) were found as independent risk factors of AECOPD >3 episodes/year. Requirement for ICU admission, invasive ventilation, and mortality rate due to AECOPDs was similar between eosinophilic and noneosinophilic groups.

**Conclusion:**

Eosinophilia upon COPD diagnosis is a factor of recurrent AECOPDs. To reduce the risk of AECOPDs and the burden of disease, clinicians may consider inhaler corticosteroids and domiciliary oxygen with a lower threshold for eosinophilic-COPD patients regardless of their clinical status.

## 1. Introduction

Chronic obstructive pulmonary disease (COPD) was reported as the third etiology of the global mortality in 2020 [1]. COPD, as a preventable and manageable chronic disorder, characterized by long-term respiratory symptoms consistent with airflow obstruction following pathophysiological irregularities of the airways or alveolar often resulting from remarkable exposures to toxic gasses or particulates over years [2]. COPD patients occasionally experience episodes of acute deterioration of respiratory status such as severe dyspnea as an acute exacerbation of COPD (AECOPD) and mainly require critical care strategies [3]. AECOPD is related to elevated risk of morbidity and in hospital mortality, as well as remarkable negative impacts on the patients' quality of life [4]. AECOPDs are relatively complex heterogeneous events for which several studies have been conducted to better understand the related clinical and paraclinical correlations [5]. More specifically, serum biomarkers and plausible correlations with AECOPDs have been recently of interest [6]. For instance, whilst hypereosinophilia in the airway lumens is basically known to be the characteristic of asthma, recent studies have suggested that sputum eosinophilia could be associated with a particular inflammatory phenotype of COPD patients [7]. It could be expected that the percentage of peripheral blood eosinophils could be a biomarker that reflect the bronchial eosinophilia with reasonable sensitivity [8]. The measurement of blood eosinophils in COPD patients carried out either in the stable state or during an exacerbation event could be a clue of the patients' status [9]. For instance, it is shown that the high blood eosinophil levels are independent prognosticator of mortality in AECOPD patients complicated by pneumonia [10]. Furthermore, it was revealed that COPD patients with eosinophilia had more chance of survival upon AECOPDs as they had better response to systematic corticosteroids [11, 12]. Herein, the aim of our study was (1) to evaluate whether the eosinophil counts could have a correlation with frequency of AECOPD episodes in newly diagnosed COPD patients and (2) to evaluate the blood eosinophil as a prognosticator of AECOPD clinical outcomes and compare it with other well-acknowledged risk factors.

## 2. Materials and Methods

### 2.1. Study Design and Participants

**Figure 1:**
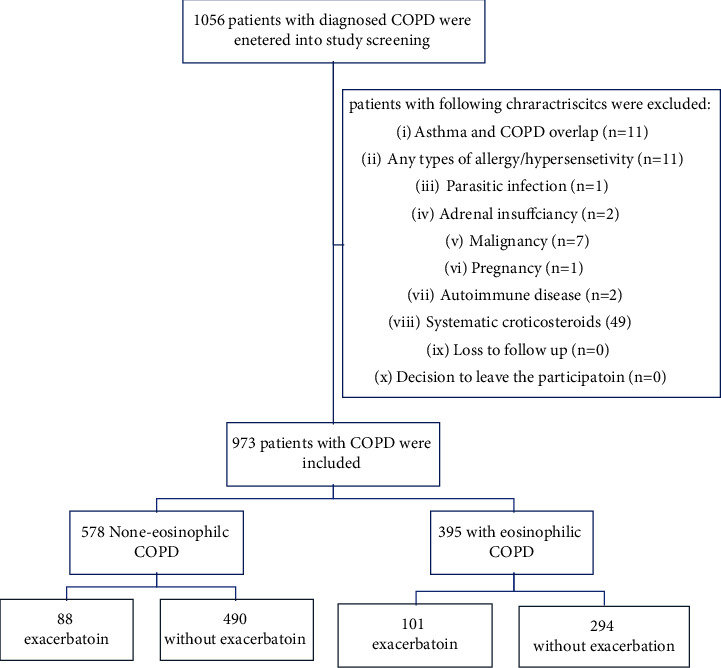
Fellow-chart for the study design. COPD = chronic obstructive pulmonary disease.

This observational, prospective, longitudinal monocentric study, evaluated new-diagnosed cases of COPD referred to the pulmonology department at the Masih Daneshvari Hospital in Tehran, Iran, from 1st March 2020 until 1st March 2022 (Figure 1). Masih Danshvari Hospital is one of the major referral pulmonology centers in the middle-east region with a remarkable load of emergent cases such as AECOPDs. The subjects included in this study were new COPD cases who were diagnosed based on the presence of the following criteria: (1) respiratory symptoms suggestive of COPD diagnosis (dyspnea, exertional dyspnea, and productive cough) for exceed of 3 months in previous two consecutive years with no other etiologies founded in evaluations, (2) significant exposure to toxic particles, particularly, history of smoking ≥10 pack-years, and (3) presence of suggestive spirometry pattern of postbronchodilator forced expiratory volume in the first second (FEV1) to forced vital capacity (FVC) ratio less than 70% (FEV1/FVC < 200 cells/microliter). Patients who had each of the following mentioned criteria were excluded from the analysis: (1) Asthma and COPD overlap, (2) any types of allergy/hypersensitivity, (3) parasitic infection, (4) adrenal insufficiency, (5) malignancy, (6) pregnancy, (7) autoimmune diseases, and (8) receiving systematic corticosteroids (Figure 1). Primary eosinophilic count for each patient was extracted from CBC diff upon the diagnosis of COPD, and we divided the patients into 2 groups: eosinophilic patients (eosinophils ≥200 cells/microliter) and noneosinophilic patients (eosinophils <200 cells/microliter). Data regarding sociodemographic (gender, age, status, pack-year of smoking, and body mass index (BMI)), comorbidities, influenza and pneumococcal vaccination, clinical (based on the global initiative for chronic obstructive lung disease (GOLD) score and COPD assessment test (CAT) score questionnaires), biological (full blood count, blood gas, and spirometry), paraclinical (Cor pulmonale, pulmonary hypertension (HTN), coronary artery diseases (CAD), diabetes mellitus (DM), dyslipidemia, and HTN), medications for COPD (bronchodilators, inhaling corticosteroids (ICS), domiciliary oxygen, and rehabilitation), and clinical sequels (AECOPDs frequency, length of hospitalization, intensive care requirement, and mortality rate) were recorded in a computer-based online system designed by our pulmonology research.

### 2.2. Definitions

“AECOPD” episode was defined as an acute exacerbation of respiratory status in a patient diagnosed with COPD requiring at least 24 hours of hospitalization. The diagnosis of ACECOPD was considered after excluding other differential diagnosis such as acute heart failure, arrhythmias, strokes, pulmonary embolism, and even psychological disorders. “Annual exacerbation” determined as the numbers of the AECOPDs episodes from the initial diagnosis of COPD until 1 year later. “CAT score” is a scoring scale to assess the life quality of COPD patient by evaluating the frequency of cough, presence of mucosa, tightness feeling in chest, exertional dyspnea, daily activities, and sleep quality. Each question possesses 0 to 5 points and eventually a patient scores 0 to 40 points based on his answers. The greater score indicates more severe COPD and the lower quality of life. “GOLD score” is an estimator of 3-year mortality for COPD patients using the CAT score, FEV1/FVC level, and number of annual exacerbations. The results categorized into 4 stages where higher stage indicated the higher risk of morbidity and mortality. “Annual mortality” determined as the event of death for the patient from the initial diagnosis of COPD until 1 year later. “Duration of hospitalization per exacerbation” determined as the average days of hospital admission due to exacerbation. Of instance, if a patient had 2 episodes of exacerbation with 2- and 4-days length of stay, we considered him as 3 days for duration of hospitalization per exacerbation. “Annual hospital stays” determined as total duration of hospital stays in emergency department, wards, or intensive care units for cardio-pulmonary status deterioration due to baseline COPD. “Cor pulmonale” as a right ventricular dysfunction due to prolonged pulmonary hypertension was diagnosed based on abnormal heart rhythms, right ventricular hypertrophy signs in radiologic chest computed tomography (CT) scan, electrocardiography, and echocardiography as well as evidence of fluid retention, and protruding neck veins. Some patients also (48%) had right heart catheterization as a gold standard of Cor pulmonale diagnosis. All COPD patients diagnosed in our center received echocardiography, hence, screening the pulmonary artery hypertension and Cor pulmonale. “Body mass index” was measured using BMI = weight (kg)/height (m)^2^.

### 2.3. Statistical Analysis

Categorial and continuous variables were presented as a case number (percentage) and the mean ± standard deviation, respectively. All data were analyzed to be confirmed as being normally distributed by the Shapiro–Wilk test. To compare the categorical variables, chi-squared or Fisher's exact test was utilized as appropriate. Independent *t*-test or Mann–Whitney *U* test was used for comparison of the mean between the continuous variables as necessitated. Data with skewed distribution were shown as the median (IQR) and comparisons were performed by Kruskal–Wallis test. Since the study was conducted on the prospective design, the Cox regression model was preferred rather than the logistic regression tests to evaluate the risk impact of predictors in the occurrence of annual AECOPDs. Primarily, we conducted the univariate Cox regression to identify the predictors of AECOPDs. Those with statistical (*p* value <0.1) and clinical importance were entered in the multivariate Cox proportional regression. We designed three multivariate models to reveal the risk factors of the AECOPD episodes >1 events/year, >2 events/year, and >3 events/year. Since the medications such as inhaling corticosteroids may affect the number of eosinophils and clinical end-points, we adjusted all of our study models for COPD medications (inhaling corticosteroids (ICS), bronchodilators, domiciliary oxygen, and rehabilitation). To evaluate the correlation of blood eosinophilic count with the number of AECOPDs in a year, a polynomial regression model was performed. A linear regression model was also designed to estimate the association between the number of eosinophils and further risk of AECOPDs. In order to assess the predictive value of eosinophilic counts for AECOPDs in different cutoffs (300, 600, and 900 cells/microliter), we conducted receiver operation curves. CAT score and GOLD score were calculated for each patient upon COPD diagnosis using online MedCalc statistical software which has continuing medical education (CME) credit. A two-sided *p* value <0.05 was deemed as significant in analyses for this study. All the statistics were performed using IBM SPSS version 27.0 software (Chicago, Illinois, US).

### 2.4. Ethical Considerations

The initial proposal and final essay was reviewed and approved by the Ethical Commitment of Shahid Beheshti University of Medical Sciences in Iran (ethical number: IR.SBMU.MSP.REC.1399.272). All participants in this study fulfilled a letter of contest for cooperating this study. All procedures of this study were performed based on the ethical guidelines of the 1975 Helsinki Declaration.

## 3. Results

### 3.1. Baseline Sociodemographic and Clinical Characteristics

**Figure 2:**
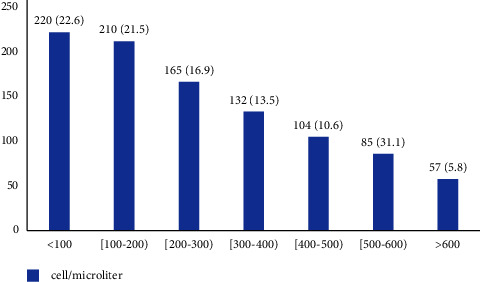
Distribution of COPD patients according to the blood eosinophilia rate upon diagnosis.

In our study, we included a total of 973 newly diagnosed COPD patients who were categorized into two groups eosinophilic (*n* = 395) and noneosinophilic (*n* = 578) using blood eosinophil = 200 cells per microliter as a cutoff point (Figure 1). Of the total, about 60% (595/973) of the patients had absolute eosinophilic counts 600 cells/microliter (Figure 2). The two groups had similar sociodemographic status in predominancy of the male gender, mean age of sixties, with overweight BMI (Table 1). Eosinophilic patients significantly smoked more (79.3% (313/395) vs. 73.5% (425/578), *p* value = 0.04) with higher severity (pack-year: 22.3 ± 5.0 vs. 17.2 ± 4.8, *p* value = 0.00). Whilst pulmonary hypertension was found more in eosinophilic COPD (25.5% (101/395) vs. 20.0% (116/578), *p* value = 0.04), the coexistence of other comorbidities such as Cor pulmonale, HTN, CAD, and DM had a similar distribution in the groups. The two groups had also a non-significant difference in spirometry test reports such as FEV1 (73.8 ± 2.4 vs. 74.1 ± 2.0, *p* value = 0.021), FEV1 to FVC ratio (66.8 ± 2.8 vs. 67.0 ± 3.0, *p*-value = 0.19), and the levels of improvement in FEV1 following usage of bronchodilator (8.4 ± 1.6 vs. 8.7 ± 1.9, *p* value = 0.31) (Table 1). To evaluate the COPD severity of the patients we conducted the CAT and GOLD scores for the patients whom most of them had relatively mild to moderate severity status; we found that 89.8% (355/395) eosinophilic and 89.1% (515/578) noneosinophilic patients had GOLD II score (*p* value = 0.77); as well as 69.3% (274/395) and 72.7% (420/578) eosinophilic and noneosinophilic groups, respectively, had the CAT score between 10 to 20 as a mild impact level (*p* value = 0.24) (Table 1). Prescribed COPD inhaler medications such as short/long beta-agonists, anticholinergics, and ICS were similar in both the groups. Also, a number of patients who received rehabilitation strategy and domiciliary oxygen support were similar in both the groups. Eventually, no statistical difference was found for a history of pneumococcal (50.6% (200/395 vs. 48.8 (2882/578), *p* value = 0.58) or influenza (4.3% (17/395) vs. 4.4% (26/578), *p* value = 0.58) vaccinations between the groups (Table 1).

### 3.2. Primary Blood Eosinophils Association with Frequency of Exacerbations

**Figure 3:**
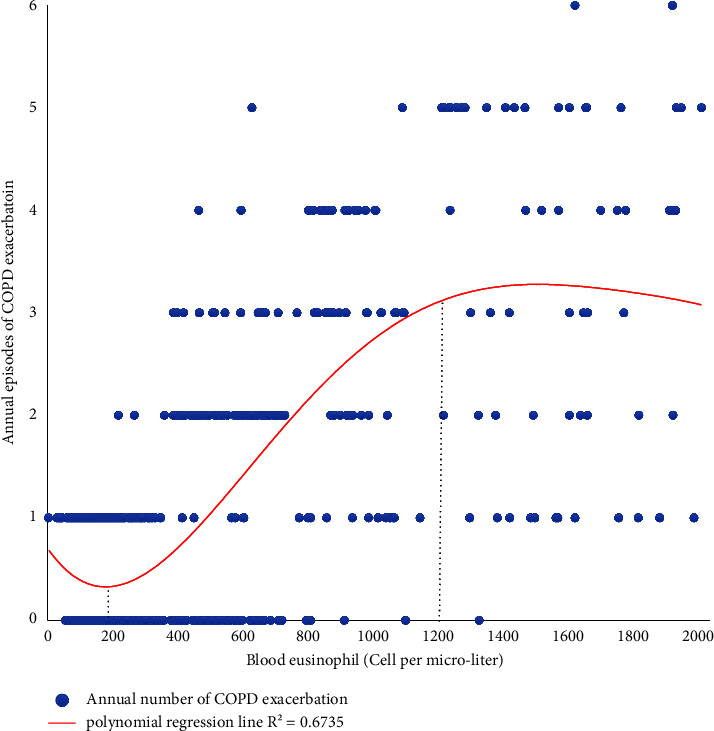
Correlation of blood eosinophilic count with episodes of COPD within a year using the polynomial regression model adjusted for COPD medications (beta agonist, anticholinergics, inhaler corticosteroids, and home oxygenation support).

**Figure 4:**
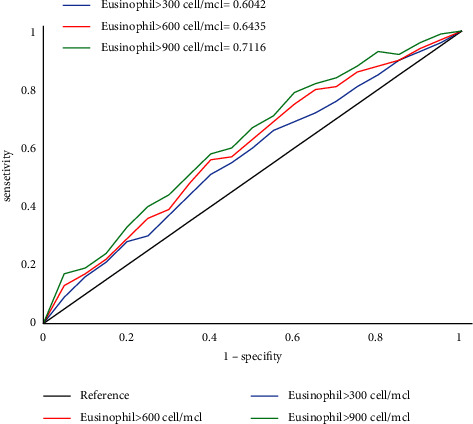
Predictive value of serum eosinophilic count for the annual COPD exacerbation ≥1 episode using area under receiver operating curve (AUROC). COPD = chronic obstructive pulmonary disease.

Using the polynomial regression model, we found that as the number of absolute blood eosinophils increased from 200 to 1,200 cells/microliter, the events of AECOPDs raised a strong positive correlation (*R*-squared = 0.6735) (Figure 3). Due to the linear relation for an eosinophilic count between 200 and 1,200 cell/microliters and annual exacerbation, we used further the linear regression model (Table 2). Accordingly, by each increase for about 180 cells/microliter, the patient experienced one more exacerbation for the annual exacerbation even when we controlled the effect of COPD-related medication.  Table 3 depicts the diagnostic accuracy of eosinophilic count for incident COPD in newly diagnosed patients by 100 intervals (cell/microliter). Accordingly, the eosinophilic count of 800 cells/microliter had the largest Youden index (0.596) with sensitivity and specificity of 80.2% and 76.6%, respectively. To estimate the predictive value of blood eosinophil count on prognosticating occurrence of the annual AECOPD (with frequency ≥1 episode), we deigned ROC for 3 cutoff points of blood eosinophils (300, 600, and 900 cells per microliter). The eosinophil >900 cells/microliter had highest sensitivity (area under ROC (AUROC) = 71%). Eosinophil >600 cells/microliter and eosinophil >300 cells/microliter had sensitivity of 64% and 60%, respectively (Figure 4). The Cox proportional hazard model was conducted to estimate the hazard ratio of eosinophilic count and other well-acknowledged risk factors of the annual COPD exacerbation as comparisons (Table 4). We enrolled male gender, BMI, active smoking, the severity of smoking (pack-year), FEV1/FVC, CAT score, GOLD score, pulmonary HTN, annual influenza vaccination, leukocytosis, and blood eosinophils variables as plausible risk factors of annual AECOPDs in the univariate model. Of these, male gender (HR = 1.30, 95% CI = 1.26–3.24, *p* value = 0.02), FEV1/FVC (HR = 1.42, 95% CI = 1.13–2.65, *p* value = 0.00), Gold score (HR = 1.35, 95% CI = 1.12–2.10, *p* value = 0.03), not receiving influenza (HR = 1.41, 95% CI = 1.16–2.35, *p* value = 0.04) or pneumococcal vaccination (HR = 1.44, 95% CI = 1.21–2.26, *p* value = 0.01), and primary eosinophils (HR = 1.92, 95% CI = 1.39–2.51, *p* value = 0.00) were known as significant risk factors. Based on the multivariate Cox regression, EV1/FVC (HR = 1.52, 95% CI = 1.18–2.75, *p* value = 0.00), GOLD score (HR = 1.32, 95% CI = 1.01–2.79, *p* value = 0.00), not receiving pneumococcal vaccination (HR = 1.35, 95% CI = 1.20–2.62, *p* value = 0.01), and primary eosinophils (HR = 1.92, 95% CI = 1.39–2.51, *p* value = 0.03) were found as prognosticators of annual exacerbation occurrence (Model 1, Table 4). In a complex Cox model, we revealed FEV1/FVC (HR = 1.61, 95% CI = 1.11–2.03, *p* value = 0.00), GOLD score (HR = 1.39, 95% CI = 1.09–2.40, *p* value = 0.00), and primary eosinophils (HR = 1.65, 95% CI = 1.41–2.29, *p* value = 0.04) as factors leading to more than 2 episodes of exacerbation per year (Model 2, Table 4). Eventually, only blood eosinophilic numbers (HR = 1.44, 95% CI = 1.33–2.15, *p* value = 0.03) and GOLD score (HR = 1.19, 95% CI = 1.30–1.52, *p* value = 0.03) were recognized as independent risk factors of the annual COPD exacerbation with more than 3 episodes in one year (Model 3, Table 4). As it is shown in Table 5, 25.6% (101/395) of eosinophilic patients suffered from COPD exacerbation in one year follow-up, which was considerably higher (*p* value = 0.01) compared to noneosinophilic patients (15.2%, (88/578)). Also, eosinophilic cases remarkably experienced more episodes of the annual exacerbation (2.26 ± 1.3 vs. 1.23 ± 0.3 episodes, *p* value = 0.00) and subsequently more total days of being admitted in hospitals in a year (9.2 ± 3.1 vs. 3.3 ± 1.6 days, *p* value = 0.00). Eosinophilic cases faced exacerbation an average of about every 136 days which was a remarkably shorter interval compared with noneosinophilic patients who experienced AECOPD every 197 days (*p* value = 0.00).

### 3.3. Primary Blood Eosinophils Association with Severity of Exacerbations

As it is shown in Table 5, we compared the patient's status and clinical end-points due to COPD exacerbation between eosinophilic and noneosinophilic patients. The gasometrical parameters assessed upon admission in the emergency department, including PaO_2_, PaCO_2_, and pH were similar between the groups, although eosinophilic cases had remarkably lower oxygen saturation (91.1 ± 3.6 vs. 92.6 ± 3.7, *p* value = 0.03). Also, inflammatory markers (qualitative CRP and ESR) and the levels of white blood cells had no significant difference between the groups (Table 5). Even though bacterial or viral pneumonia had similar responsibility for the occurrence of COPD exacerbations, patients in the eosinophilic group suffered more from bacterial and viral coinfection pneumonia (6.1% (24/395) vs. 3.1% (18/578), *p* value = 0.02). Notwithstanding, the rate of ICU admission, the requirement for invasive respiratory support, duration of hospital stay per exacerbation, and fatality rate had not statistically significant difference among the groups (Table 5).

## 4. Discussion

Our study aimed to investigate the possible association of the initial blood eosinophilia levels with the frequency and severity of annual AECOPDs. We found that (1) the levels of peripheral eosinophils were not associated with patients' initial clinical status, which noneosinophilic and eosinophilic groups had similar respiratory function as well as similar CAT and GOLD scores upon the diagnosis; (2) eosinophilic patients smoked more with higher severity and also had more prevalence of pulmonary hypertension. Accordingly, the relation of the intensive and chronic history of tobacco use with increasing bronchial eosinophilic count has been previously reported [9, 13]; (3) the initial eosinophilic count could forecast the risk of annual AECOPDs with high sensitivity in which those who had higher levels of blood eosinophils upon the COPD diagnosis, had higher chance of recurrence in AECOPDs episodes; (4) finally, we revealed that whilst patients with primary eosinophilic number confront more episodes of exacerbation, they suffered similar clinical severity, hospital duration, and final outcome in each exacerbation compared to the noneosinophilic ones. Indeed, the increase in the rate of initial eosinophils in COPD patients does not seem to have a considerable influence on AECOPD clinical outcomes according to the results of our study.

To support our findings, it is revealed that eosinophilic bronchial inflammation is associated with increasing episodes of COPD exacerbations [14]. Similarly, a reduction in eosinophils in the sputum by ICS was shown to be associated with a reduction in the frequency of exacerbations of COPD [15–17]. Vedel-Krogh et al. [15] in a cohort of 203 patients suffering from COPD in Denmark found that there was a higher rate of exacerbations during 5 years in patients with the eosinophil levels >2% at a stable clinical state. Also, the results of a population-based analysis on 2,146 COPD patients in the United States, a linear relation between the number of absolute sputum eosinophils with the episodes of AECOPDs have been found [16]. In other evaluation by Couillard et al. showed the risk of readmission in 12-months was higher for COPD patients with blood eosinophilia at first admission ≥200 cells/microliter or ≥2% [17]. Moreover, in a meta-analysis by Ho et al. [18], eosinophilic COPD patients had a shorter interval for the occurrence of the next exacerbation, though such patients had a better quality of life and better response to inhaled corticosteroid therapy compared to noneosinophilic ones. Notwithstanding, we did not find an association between eosinophils and severity of the AECOPD episodes, particularly in terms of the gasometrical data, the length of hospital stays or requirement for intensive mechanical ventilation, and mortality between two groups. Similar to our findings, in a multicenter randomized controlled trial conducted by Bafadhel et al. [19] on 243 COPD patients hospitalized for AECOPD, eosinophilic COPD patients (eosinophils >200 cells/microliter), had no longer had hospital stays compared to the group of noneosinophilic patients. Also, other studies reported that the concentration of sputum eosinophils does not have an effect on the length of the hospital stay [20], or the requirement for ICU admission during AECOPD [21]. Of interest, it is reported an inverse relationship between blood eosinophil counts and bacterial infection in patients with COPD [8]. Kolsum et al. [8] found that COPD cases with lower absolute blood eosinophils faced more complicated bacterial pneumonia. Since bacterial infections could be potentially linked with deterioration of COPD patient clinical status and even occurrence of AECOPDs, it could be interpreted that the relationship between the eosinophil levels and the severity of the exacerbation episodes could be even reversed. As we showed, high numbers of upon-diagnosis blood eosinophils did not increase the likelihood of the annual mortality in our COPD patients. Near to our findings, in the study by Bafadhel et al. [19], the annual mortality rate was similar between the two eosinophilic and noneosinophilic groups. Other longitudinal cohort studies reported similar three-year survival between the groups of eosinophilic and noneosinophilic patients and also among patients with high and very high eosinophil thresholds (2%, 3%, and 4% blood eosinophils) [22]. Furthermore, a recent study found that the decrease of blood eosinophils to a lower number of 150 cells/microliter, could be an independent risk factor of 9-year mortality in COPD [10]. Eventually, some questions remained to be asked in this aspect; by reviewing the previous literature, it is obtained that there is neither consensus on the use of absolute or relative blood eosinophil counts nor on the recommended circulating eosinophil count cutoffs for the best comparative results. Thus, different thresholds are applied in the studies making the literature heterogeneous [22–24]. Moreover, whilst there are some reports regarding the relation of sputum or the blood eosinophil levels upon the exacerbation with clinical outcomes, the literature data evaluating the primary eosinophil levels just after diagnosis, stable states, or overtime fluctuation of this biomarker is extremely scarce. Concerning the discordance of the data of our study and others in terms of the correlation between eosinophils and the aggravation of the risk during severe AECOPDs, more exploration of the predictive value of this biomarker seems necessary. Other studies targeting eosinophil counts in COPD patients with the stable conditions to assess leading sequels of disease like AECOPDs are required for better assessment of the prognosis and follow therapeutic decisions.

### 4.1. Strengths and Limitations

We evaluated the association of eosinophilic counts upon diagnosis of COPD in cases who were clinically stable to forecast the probity of annual exacerbations in such patients; in contrast, most of the previous studies considered the number of eosinophilic counts upon exacerbation when the patients were in a critical status. Taking into account this point, this essay was a novel analysis in the field of the COPD survival analysis. Furthermore, the heterogenicity of the patients and the size of the study population due to conducting this essay in a major referral center was the strength of our study. Also, we performed our analysis in a prospective longitudinal aspect in contrast to most of the similar studies that retrospectively evaluated the role of biomarkers on COPD prognosis. We also utilized various models of statistics to better estimate the blood eosinophils as predictors of AECOPDs. Also, even though some studies did not exclude the asthma-overlap patients, we excluded such patients to reduce the misclassification and increase the accuracy of the results. The exclusion of asthma-COPD patients was based on the clinical judgment of experienced pulmonologists, although we did not separately conduct IgE or allergic-related laboratorial essays. The most common patients who were diagnosed as asthma-COPD overlap were moderate smokers with prior asthma who had non-fully reversible airflow obstruction in spirometry and had an onset of symptoms during their late 30 s or early 40 s. Also, patients with strong favorable history for diagnosis who had a promising response to bronchodilators in spirometry were considered as asthma-COPD cases. Although we believe that exact diagnosis of such patients might be challenging, we could at least in part reduce the confounding effect of such patients in our study.

Our study had some limitations worth to be discussed: first, we evaluated the peripheral eosinophilic count and not the sputum concentration of the airway eosinophils which could be more precisely correlated with AECOPDs. Second, we did not include patients receiving the systematic corticosteroids and also adjusted our analytic models with other COPD medications (bronchodilators, ICS, rehabilitation, and domiciliary oxygenation) to restrict the impact of therapeutics on the pure effect of eosinophils on AECOPDs. Hence, the impact of corticosteroids on AECOPDS could be modest which required more comparative studies. Notably, most of the patients included in our study had mild severity of disease in both the groups (GOLD II: 89.8% vs. 89.1% *p*=0.72) and 80.5% of them did not either expertise annual exacerbation. This could explain why only 7.8% of our patients received ICS adjuvant therapy. Third, also we considered all the well-acknowledged risk factors of COPD exacerbation in our Cox hazard model based on our available data, it was not possible for us to enroll all risk factors of exacerbation such as exposure to ambient air pollution, history of childhood pneumonia, or Alph-1 deficiency. Last but not the least, although most of our cases with AECOPDs were referred to our emergency department, there were also few self-reports (0.7%) of hospitalization due to AECOPDs in other centers. Though the documents of such hospitalizations were thoroughly considered, there would be a rare but possible probability that AECOPDs in other centers be diagnosed with different criteria.

## 5. Conclusion

The main conclusion drawn from this study is the strong predictive value of the initial blood eosinophilic count on the increasing risk of annual COPD exacerbation events. We have shown the number of initial eosinophils that has a relative linear correlation with the number of the exacerbation episodes. Meanwhile, an eosinophilic count >200 cells/microliters do not change the prognosis of COPD exacerbations in most clinical, biological, and survival characteristics compared to lower amounts of peripheral eosinophils. Therefore, initial therapeutic strategies in order to mitigate the number of the annual exacerbation episodes should be conducted in eosinophilic patients. Of instance, the pulmonologist may be having fewer thresholds to initiate ICS or even oral corticosteroids as well as daily oxygen supports in-house (domiciliary oxygenation) as established promising options reducing the number of AECOPDs in such patients. Notably, despite the suggestions for considering the eosinophil levels in therapeutic decisions in recent years, the prognostic value of blood eosinophils in COPD as a population-dependent marker should not be neglected. Indeed, in the phenotyping evaluation of COPD patients, different environmental variables also should be taken into account. To our knowledge, this was the first assessment regarding this aspect conducted on a middle-eastern population in Iran. Further studies are needed for a better evaluation of this phenotyping biomarker for COPD patient prognosis.

## Figures and Tables

**Table 1:** Epidemiological and clinical characteristics of the patients diagnosed with COPD.

Variables	Eosinophilic *n* = 395	Noneosinophilic *n* = 578	*p* values
*Sociodemographic*			
Age (year)	63.4 ± 5.3	63.9 ± 4.1	0.09
Sex (male)	301 (76.2)	463 (80.1)	0.14
BMI (kg/m^2^)	29.2 ± 3.1	29.0 ± 3.7	0.37
Active smoker	313 (79.3)	425 (73.5)	**0.04**
Pack-year in active smoker	22.3 ± 5.0	17.2 ± 4.8	**0.00**
Former smoker	20 (5.0)	32 (5.5)	0.73

*Comorbidities*			
Pulmonary hypertension (%)	101 (25.5)	116 (20.0)	**0.04**
Cor pulmonale	71 (17.9)	82 (14.2)	0.12
Hypertension	255 (64.5)	395 (68.3)	0.22
Coronary artery disease	121 (30.6)	179 (30.9)	0.92
Diabetes melilotus	65 (16.4)	87 (15.0)	0.55
Dyslipidemia	199 (50.4)	254 (44.0)	0.05

*Spirometry*			
FEV1 (%)	73.8 ± 2.4	74.1 ± 2.0	0.21
FEV1/FVC	66.8 ± 2.8	67.0 ± 3.0	0.19
FEV1 improvement after bronchodilator	8.4 ± 1.6	8.7 ± 1.9	0.31

*COPD severity score*			
CAT score (impact level)	13.8 ± 2.3	13.6 ± 2.1	0.16
Low (<10)	54 (13.7)	58 (10.0)	0.07
Medium (10–20)	274 (69.3)	420 (72.7)	0.24
High (21–30)	45 (11.4)	59 (10.2)	0.55
Very high (>30)	22 (5.6)	41 (7.1)	0.35
GOLD categorization	2 (2.3)	2 (2.3)	0.56
I	28 (7.1)	44 (7.6)	0.77
II	355 (89.8)	515 (89.1)	0.72
III	7 (1.7)	9 (1.5)	0.81
IV	5 (1.3)	10 (1.7)	0.61

*COPD therapeutics*			
Short-acting beta agonist	395 (100)	578 (100)	1.00
Long-acting beta agonist	374 (9.5)	562 (9.7)	0.91
Anticholinergics	155 (39.2)	216 (37.4)	0.57
Inhaled corticosteroids	29 (7.3)	47 (8.1)	0.64
Home oxygenation support	35 (8.9)	41 (7.1)	0.30
Rehabilitation	65 (16.4)	100 (17.3)	0.71

*Prophylactic vaccination*			
Pneumococcal vaccination	200 (50.6)	282 (48.8)	0.58
Influenza vaccination	17 (4.3)	26 (4.4)	0.94

COPD = chronic obstructive pulmonary diseases.

**Table 2:** Association of an annual COPD exacerbation with the blood eosinophils level for patients with baseline serum eosinophil 200–1,200 cell per microliter using the linear regression model.

*Model 1*		*Model 2*	
Beta (95% CI)	*P* value	Beta (95% CI)	*P* value
184 (156–285)	<0.001	179 (153–294)	0.008

Beta shows the number of eosinophilic counts (cell/microliter) increased by each annual exacerbation. Model 1: unadjusted. Model 2: further adjusted for COPD medications (bronchodilators, inhaler corticosteroids, domiciliary oxygen, and rehabilitation). COPD = chronic obstructive pulmonary disease.

**Table 3:** Diagnostic accuracy of eosinophilic count for incident COPD in newly diagnosed patients by 100 intervals (cell/microliter).

Cutoff	Sensitivity	Specificity	Youden index
100	98.6	21.9	0.226
200	97.5	33.8	0.274
300	97.2	42.4	0.295
400	97.0	49.9	0.348
500	88.8	56.7	0.423
600	85.2	63.6	0.517
700	83.9	70.4	0.526
800	80.2	76.6	0.596
900	77.6	82.3	0.546
1,000	75.2	87.8	0.523
1,100	71.0	92.6	0.501
1,200	70.3	95.3	0.485
1,300	68.4	96.5	0.425
1,400	69.5	96.9	0.412
1,500	66.7	97.1	0.406

COPD = chronic obstructive pulmonary disease.

**Table 4:** The Cox proportional hazard model for evaluating the risk of annual exacerbation adjusted for COPD medications (bronchodilators, inhaler corticosteroids, domiciliary oxygen, and rehabilitation).

*Variables*	Units/levels	*Univariate*		*Multivariate*					
				*Model 1*		*Model 2*		*Model 3*	
Events		*Annual exacerbation (any)*		*>1 annual exacerbation*		*>2 annual exacerbations*		*>3 annual exacerbation*	
		HR (95% CI)	*P* value	HR (95% CI)	*P* values	HR (95% CI)	*P* values	HR (95% CI)	*P* values
Gender	Male vs. female	1.30 (1.26–3.24)	**0.02**	1.14 (1.00–1.44)	**0.09**	1.23 (0.92−0.41)	0.21	1.13 (0.81–1.85)	0.29
BMI	Each 1 kg/m^2^ increase	1.12 (0.92–1.58)	0.22	—	—	—	—	—	—
Smoke	Presence vs. absence	1.45 (0.88–4.62)	0.19	—	—	—	—	—	—
Pack-year	Each 10-unit increase	1.12 (1.00–1.96)	0.07	—	—	—	—	—	—
FEV1/FVC	Each 10-unit decrease	1.42 (1.13–2.65)	**0.00**	1.52 (1.18–2.75)	**0.00**	1.61 (1.11–2.03)	**0.00**	1.33 (0.90–2.63)	0.21
CAT score	Each 5-unit increase	1.23 (0.92–1.65)	0.25	—	—	—	—	—	—
Gold score	≥3 (3, 4) vs. <3 (1, 2)	1.35 (1.12–2.10)	**0.03**	1.32 (1.01–2.79)	**0.00**	1.39 (1.09–2.40)	**0.00**	1.19 (1.30–1.52)	**0.03**
Cor pulmonal	Presence vs. absence	1.23 (0.90–2.63)	0.17	—	—	—	—	—	—
Influenza vaccination	Absence vs. presence	1.41 (1.06–2.35)	**0.04**	1.31 (0.91–2.41)	0.08	1.21 (0.84–2.12)	0.25	1.29 (0.79–1.82)	0.21
Pneumococcal vaccination	Absence vs. presence	1.44 (1.21–2.26)	**0.01**	1.35 (1.20–2.62)	**0.09**	1.25 (0.88–2.63)	0.30	1.31 (0.92–1.51)	0.21
Leukocytosis	Presence vs. absence	1.09 (1.00–1.13)	0.35	—	—	—	—	—	—
Eosinophils	Each 50 cell/microliter increase	1.92 (1.39–2.51)	**0.00**	1.92 (1.39–2.51)	**0.03**	1.65 (1.41–2.29)	**0.04**	1.44 (1.33–2.15)	**0.03**

*P* value <0.1 are bold in univariate analysis column. *P* value <0.05 are bold in multivariate analysis columns. Variables with *p* value <0.1 were entered into the multivariate model. The role of each variable in different cutoff episodes of exacerbation has been evaluated in 3 multivariate Cox models.

**Table 5:** Clinical and laboratorial characteristics of the patients admitted in emergency department whilst exacerbation of COPD.

Variables	Eosinophilic *n* = 395	Noneosinophilic *n* = 578	*p* values
*Gasometrical*			
PaO_2_ (mmHg)	61.2 ± 18.2	59.9 ± 15.4	0.38
PaCO_2_ (mmHg)	39.2 ± 5.9	38.8 ± 5.6	0.23
pH	7.25 ± 2.6	7.30 ± 3.5	0.09
Oxygen saturation without oxygen support (%)	91.1 ± 3.6	92.6 ± 3.7	**0.03**

*Laboratorial*			
CRP qualitative (0+ to 4+)	2 (2, 3)	1 (2, 2)	0.09
ESR (mg/L)	16 ± 5.6	15 ± 4.3	0.21
Leucocytes (cell per microliter)	6,850 (5,610, 8,560)	6,740 (5,590, 8,445)	0.09
Eosinophils (cell per microliter)	361 (210, 643)	176 (141, 352)	**0.00**

*Clinical severity*			
Only bacterial pneumonia	81 (20.5)	98 (17.0)	0.16
Only viral pneumonia	39 (9.8)	50 (8.6)	0.52
Bacterial and viral pneumonia coinfection	24 (6.1)	18 (3.1)	**0.02**
ICU-admission (yes)	100 (25.3)	142 (24.5)	0.19
Invasive mechanical ventilation (yes)	27 (6.8)	30 (5.2)	0.25
Annual exacerbation event (yes)	101 (25.6)	88 (15.2)	**0.01**
Annual exacerbation frequency (number of events)	2.26 ± 1.3	1.23 ± 0.3	**0.00**
Interval for next exacerbation (days)	135.9 ± 25.9	197.0 ± 34.0	**0.00**
Duration of hospitalization per exacerbation (days)	3.3 ± 1.3	2.8 ± 1.1	0.16
Annual hospital stays (days)	9.2 ± 3.1	3.3 ± 1.6	**0.00**
Annual mortality (yes)	10 (2.5)	13 (2.2)	0.71

*P* value <0.05 are bold.

## Data Availability

The data used to support the findings of this study are available upon request from the corresponding author. The data are not publicly available due to the privacy or ethical restrictions.

## Abbreviations

AECOPD: Acute exacerbation of COPD
BMI: Body mass index
CT: Computed tomography
CME: Continuing medical education
COPD: Chronic obstructive pulmonary disease
CAT: COPD assessment test
CAD: Coronary artery diseases
DM: Diabetes mellitus
FEV1: Forced expiratory volume in the first second
FVC: Forced vital capacity
GOLD: Global Initiative for Chronic Obstructive Lung Disease
HTN: Hypertension
ICS: Inhaling corticosteroids.
